# Supporting cancer research on real-world data: extracting colorectal cancer status and explicitly written TNM stages from free-text imaging and histopathology reports

**DOI:** 10.1136/bmjhci-2025-101521

**Published:** 2025-09-21

**Authors:** Andres Tamm, Helen J S Jones, Neel Doshi, William Perry, Jaimie Withers, Hizni Salih, Theresa Noble, Kinga Anna Varnai, Stephanie Little, Gail Roadknight, Des Campell, Sheila Matharu, Naureen Starling, Marion Teare, Algirdas Galdikas, Ben Glampson, Luca Mercuri, Dimitri Papadimitriou, Harpreet Wasan, Lauren A Scanlon, Lee Malcomson, Catherine O’Hara, Andrew Renehan, Brian D Nicholson, Jim Davies, Eva J A Morris, Kerrie Woods, Chris Cunningham

**Affiliations:** 1 Nuffield Department of Primary Care Health Sciences, University of Oxford, Oxford, UK; 2 Big Data Institute, Oxford University, Oxford, England, UK; 3 NIHR Oxford Biomedical Research Centre, Oxford, UK; 4 Oxford University Hospitals NHS Foundation Trust, Oxford, England, UK; 5 University Hospital Southampton NHS Foundation Trust, Southampton, UK; 6 Mayo Clinic, Rochester, New York, USA; 7 The Royal Marsden NHS Foundation Trust, London, England, UK; 8 NIHR Biomedical Research Centre at The Royal Marsden and The Institute of Cancer Research (ICR), London, UK; 9 NIHR Imperial Biomedical Research Centre, London, UK; 10 Imperial College Healthcare NHS Trust, London, UK; 11 The Christie NHS Foundation Trust, Manchester, England, UK; 12 NIHR Manchester Biomedical Research Centre, Manchester, UK; 13 Department of Computer Science, University of Oxford, Oxford, UK; 14 Nuffield Department of Population Health, University of Oxford, Oxford, UK

**Keywords:** Colorectal Carcinoma, Electronic Health Records, Natural Language Processing

## Abstract

**Objectives:**

The ‘tumour, node, metastasis’ (TNM) classification of colorectal cancer (CRC) predicts prognosis and so is vital to consider in analyses of patterns and outcomes of care when using electronic health records. Unfortunately, it is often only available in free-text reports. This study aimed to develop regex-based text-processing algorithms that identify the reports describing CRC and extract the TNM staging at a low computational cost.

**Methods:**

The CRC and TNM extraction algorithms were iteratively developed using 58 634 imaging and pathology reports of patients with CRC from the Oxford University Hospitals (OUH) and Royal Marsden (RMH) NHS Foundation Trusts (FT), with additional input from Imperial College Healthcare and Christie NHS FTs. The algorithms were evaluated on a stratified random sample of 400 OUH development data reports and 400 newer ‘unseen’ OUH reports. The reports were annotated with the help of two clinicians.

**Results:**

The CRC algorithm achieved at least 93.0% positive predictive value (PPV), 72.1% sensitivity, 64.0% negative predictive value (NPV) and 90.1% specificity for primary CRC on pathology reports. On imaging reports, it demonstrated at least 78.0% PPV, 91.8% sensitivity, 93.0% NPV and 80.9% specificity. For the main T/N/M categories, the TNM algorithm achieved PPVs of at least 93.9% (T), 97.7% (N) and 97.2% (M), and sensitivities of 63.6% (T), 89.6% (N) and 64.8% (M). NPVs were at least 45.0% (T), 91.1% (N), 88.4% (M), and specificities 95.7% (T), 98.1% (N), 99.3% (M). Reductions in performance were mostly due to implicit staging. For extracting explicit TNM stages, current or historical, the algorithm made no errors on 400 pathology reports and six errors on 400 imaging reports.

**Conclusion:**

The TNM algorithm accurately extracts explicit TNM staging, but other methods are needed for retrieving implicit stages. The CRC algorithm is accurate on non-supplementary reports, but outputs need additional review if higher precision is required.

**WHAT IS ALREADY KNOWN ON THIS TOPIC**

- TNM (tumour, node, metastasis) staging is fundamental to cancer classification, determining prognosis and guiding treatment decisions.
- However, unlike blood test results and demographic data, the TNM scores are often not available in a structured format within electronic health records, and the authors are not aware of any freely available tool that can extract TNM scores with sufficient flexibility and accuracy.

**WHAT THIS STUDY ADDS**

- The study provides and validates two regex-based algorithms that detect whether current primary colorectal cancer (CRC) is present, and extract the TNM staging scores given in letters and numbers from anywhere in the report, while covering variations in reporting and avoiding false positives.
- The algorithms rely on a small number of python packages and run relatively fast, which makes them easy to deploy in hospital research and information systems.

**HOW THIS STUDY MIGHT AFFECT RESEARCH, PRACTICE OR POLICY**

- The algorithms can facilitate research into CRC and help check the quality of staging data in hospital information systems.

## Introduction

 Cancer staging is essential for planning and evaluating treatment, estimating prognosis, auditing the quality of care, tracking cancers at the population level and conducting both observational and clinical research.^1^ It is most commonly recorded using the TNM classification system which has three main components^1^: T for describing the depth of the tumour and local infiltration; N for indicating if and to what extent tumour has spread to regional lymph nodes; and M for indicating if cancer has spread to another region of the body (see online supplemental materials S1). Each component is recorded using a small set of values, often reported in a sequence. For example, according to the latest eighth edition of the TNM classification by the Union for International Cancer Control (UICC), ‘T1 N0 M0’ colorectal cancer means that ‘tumour invades submucosa of the bowel (T1) and has not spread to regional lymph nodes (N0) or other organs and structures (M0).’^2^ The TNM classification also includes other categories, such as lymphatic invasion (L), venous invasion (V), perineural invasion (Pn), residual tumour status (R) and tumour grade (G), which are similarly reported with letters and numbers.

Despite being an essential descriptor of cancer, the TNM categories are not always recorded in a structured format in electronic health records. More often, they are written in free-text clinical documentation such as pathology or imaging reports. The variable ways in which they are recorded in free text can lead to false negatives when very simple pattern matching rules are used for extraction (table 1). Furthermore, clinical reports can contain alphanumeric strings that are like TNM categories but have a different meaning, which can lead to false positives. For example, 'T1' may also refer to the first thoracic vertebrae or to a T1-weighted magnetic resonance image.

**Table 1:** Variation in how TNM values may be written in free text

Example	Comment
T1/2/3 N0 V0	Multiple values given for T category
T1 N0 M0	Zero mis-spelt as O
T1 n1	Some letters written in lower case
T1N0M0	No gap between letters
T 1 N0M0	Gap between letter and value
pT1 vs ypT1 vs ymrT1	Different types of prefixes can be recorded, eg, ‘y’ for post-treatment, ‘p’ for pathological and ‘mr’ for staging based on MRI scan
T1a (solitary tumour) N0	TNM values separated by text such as comments
R0 pT3 L0 V0 N0 Mx vs pT2 N1 Mx L0 V0 R1	Multiple TNM values given in variable order
Staged as T2	Only a single TNM value given

Several attempts have been made to extract TNM staging values from free text^3^,^12^ (see the Discussion section). Most have encountered difficulties with the variation in documentation and none are freely available for public use. This study therefore set out to review the current TNM extraction tools before developing a novel regex-based natural language processing (NLP) tool that can better account for variability in TNM documentation.

## Methods

A regex-based NLP tool was developed to extract explicitly given TNM scores (TNM scores written in letters and numbers) from free-text reports, considering the variability in reporting and distinguishing TNM values from semantically different false-positive matches. The algorithm was not designed to infer implicit staging (when TNM scores are not reported). Additionally, a regex-based algorithm was developed to help find clinical reports that describe colorectal cancer (CRC), so that the TNM stages corresponding to primary CRC can be extracted from these reports. The tool is freely available at https://github.com/tammandres/crc-and-tnm. This report covers the Tripod-LLM checklist,^13^ with items specific to large language models omitted (online supplemental Materials S2).

### Iterative algorithm development on a multicentre dataset

**Figure 1:**
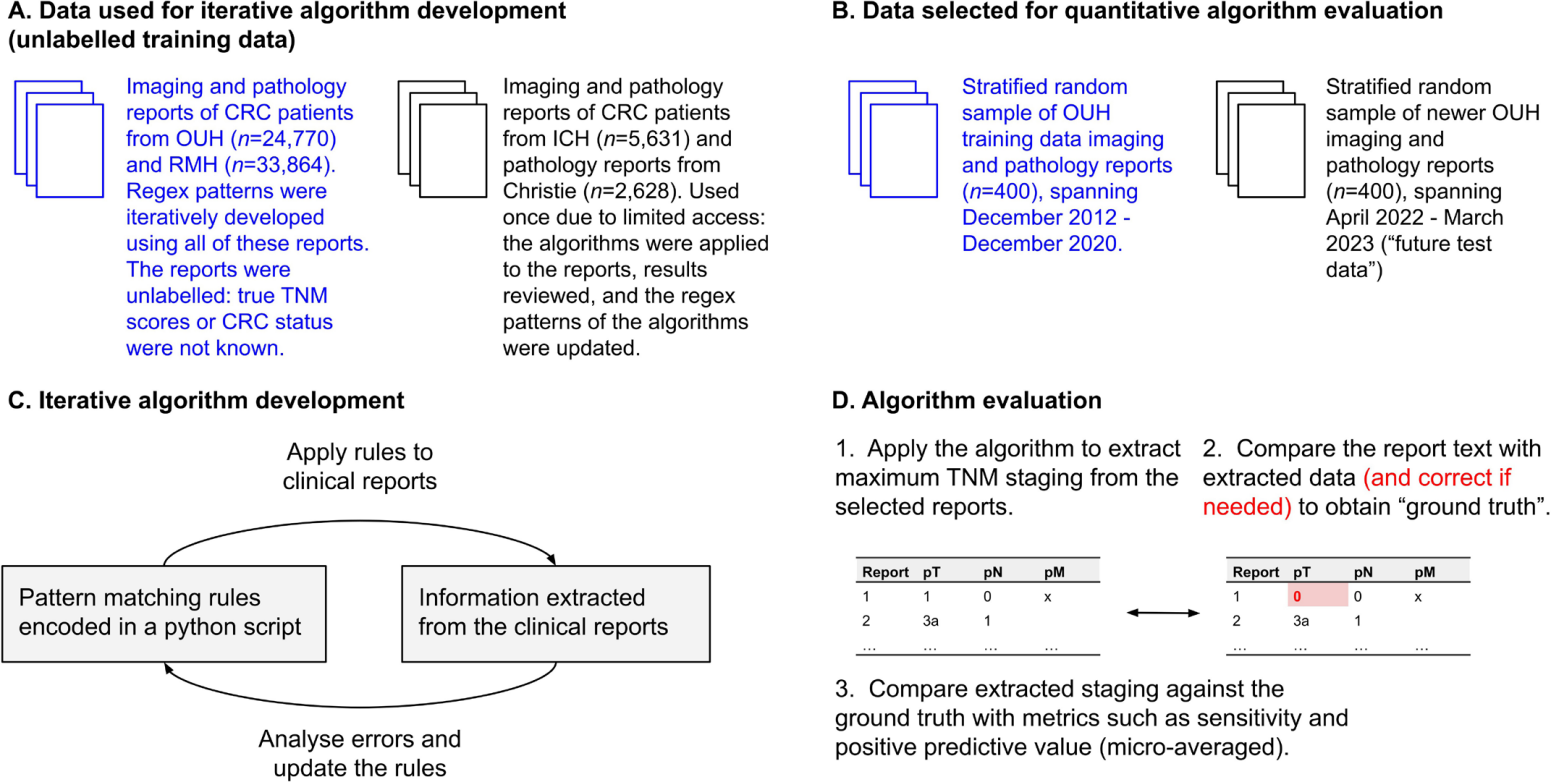
Iterative algorithm development on a multicentre dataset. CRC, colorectal cancer; ICH, Imperial College Healthcare; OUH, Oxford University Hospitals; RMH, Royal Marsden Hospital.

A CRC and TNM stage extraction pipeline was iteratively developed on imaging and histopathology reports of patients with CRC (International Classification of Diseases version 10 codes C18-C20) as part of a NIHR HIC research programme to establish a CRC database^14^ (figure 1). It was primarily developed using 10 322 imaging and 14 448 pathology reports from the Oxford University Hospitals (OUH) NHS Foundation Trust (FT), and 26 326 imaging and 7538 pathology reports from the Royal Marsden (RMH) NHS FT, a total of 58 634 reports. All of these unlabelled reports were used iteratively by generating patterns to extract the desired information (primary CRC status and TNM staging), examining the extracted matches, and then analysing errors in collaboration with clinicians. The OUH reports spanned December 2012–December 2020, and the RMH reports November 2011–September 2021. Further testing was undertaken on 4252 imaging and 1379 pathology reports from Imperial College Healthcare (ICH) NHS FT and 2628 pathology reports from the Christie NHS FT, with feedback given to the development team. Only imaging reports where the imaging type was relevant for investigating CRC were used. Sensitive information, such as patient names, was redacted with site-specific pipelines. To quantitatively evaluate the algorithms, a subset of reports was sampled for annotation (see below).

### The CRC detection algorithm

To detect whether a clinical report discusses current primary CRC, the algorithm first identified keywords that refer to colorectal tumours, and then excluded keywords that did not refer to current primary tumours based on their context. A method similar to the ConText algorithm^15^ was used for excluding keywords that were negated, historic, general, non-definite or related to metastasis, recurrence and treatment response. Extensive keywords for tumours and anatomical sites were generated with input from two authors (ND, HJSJ) (online supplemental Materials S3). When run, the algorithm keeps reports that describe CRC and outputs included and excluded phrases that contain keywords for CRC.

### The TNM stage extraction algorithm

TNM staging is commonly reported using a limited set of letters and numbers (table 2), so it should be extractable by pattern matching with regular expressions. This was accomplished by first extracting all phrases that contain TNM staging, then extracting values from the phrases. The algorithm outputs maximum scores for each TNM category and the TNM phrases. The regular expressions that extract TNM phrases were carefully designed and constrained to extract valid TNM phrases flexibly from anywhere in the report while avoiding false positives (online supplemental materials S4).

**Table 2:** TNM categories extracted by the algorithm

Shorthand	TNM category	Possible values
Tpre	Prefix of primary tumour	Combination of the letters a, c, m, p, r, y. For example, ‘yp’, ‘ymr’, ‘p’
T	Tumour extent	0, 1, 1a-1d, 2, 2a-2d, 3, 3a-3d, 4, 4a-4d, X, is
N	Nodal invasion	0, 1, 1a-1c, 2, 2a-2c, 3, 3a-3c, X
M	Distant metastasis	0, 1, 1a-1c, X
V	Venous invasion	0, 1, 2, X
R	Residual tumour status	0, 1, 2, X
L	Lymphatic invasion	0, 1, X
Pn	Perineural invasion	0, 1, X
G	Grade of differentiation	1, 2, 3, 4, X
SM	Kikuchi level*	1, 2, 3
H	Haggitt level*	0, 1, 2, 3, 4, I, II, III, IV

* The Kikuchi and Haggitt levels are not part of the TNM classification but were extracted because they are reported similarly and were of interest for colorectal cancer clinicians.

### Evaluation of the algorithms

A subset of 800 reports was randomly selected from the 24 770 OUH reports used in iterative algorithm development, and another 800 from newer unseen OUH reports. We judged whether the reports were correctly marked as describing current CRC, and whether the maximum TNM stage values were correctly extracted (as some reports contained multiple staging scores). Cross-validation was not used, because it was not feasible to repeat the iterative regex development.

#### Selection of clinical reports

To assess CRC and TNM detection, a hundred reports were randomly sampled from each of the two categories: reports where a value was detected, and reports where no value was detected. This was done separately for CRC and TNM detection, for imaging and pathology reports, and for OUH reports that were used during development (‘training data’) and a newer sample of OUH reports unseen during development (‘future test data’). Overall, this yielded 400 training data and 400 test data reports for evaluating CRC extraction, and the same number for assessing TNM extraction. The stratified random selection based on value detection helped ensure that enough positive examples were available to estimate the positive predictive value (PPV). Data from other hospitals were not used due to resource constraints. The training data reports used in evaluation spanned December 2012–December 2020, and the test data reports April 2022–March 2023.

#### Annotation of reports

The selected reports were reviewed by three authors (ND, AT, HJSJ). The initial annotations were further corrected to better include implicit staging (where staging is not given in letters and numbers but can be inferred). The algorithms were run on the selected reports and reviewed by displaying both the extracted values and reports in an R Shiny app (online supplemental figure S5). Ground truths were established by correcting extracted values, but we believe that the risk of bias was low and was outweighed by the ability to review 1600 reports with limited resources. See online supplemental materials S5 for details.

#### Performance metrics

CRC and TNM stage extraction was evaluated with the metrics of positive predictive value (PPV), negative predictive value (NPV), sensitivity and specificity. As each TNM category can take multiple values, these metrics were computed as micro-averages with reference to reports where no value was detected. PPV and sensitivity, also known as precision and recall, were the primary metrics because together they characterise the effectiveness of information retrieval.^16^ The confusion matrix for TNM scores was additionally reported. TNM stage extraction was evaluated under two scenarios. First, the retrieval of historical stages and the non-retrieval of implicit stages were considered as errors, as this would be of interest to the user. Second, as the algorithm was designed to extract explicit stages, performance was also reported without considering these as errors. See online supplemental materials S5 for more details.

### Software

The algorithms were implemented in python V.3.9 primarily using *numpy* (V.1.23.5),^17^ *pandas* (V.1.4.3)^18^ and *regex* (V.2020.10.15).^19^

## Results

### Primary CRC

The algorithm achieved 79.7% sensitivity and 94.0% PPV for detecting primary CRC in training data histopathology reports; and 94.7% sensitivity and 89.9% PPV in training data imaging reports. The NPV was 75.8% and specificity 92.6% in training data pathology reports; 95.0% NPV and 90.6% specificity were observed in training data imaging reports. Performance on future OUH reports was lower than on training data reports: on future pathology reports, the sensitivity was 72.1% and PPV was 93.0%; on future imaging reports, the sensitivity was 91.8% and PPV was 78.0%. On future pathology reports, the NPV was 64.0% and specificity 90.1%; and on future imaging reports, the NPV was 93.0% and specificity 80.9%. For confidence intervals, see table 3.

**Table 3:** Performance of a regex-based algorithm for detecting primary CRC in histopathology and imaging reports of patients with CRC in OUH

Report type	Num report	Num CRC	PPV	NPV	Sensitivity	Specificity
Subset of training data						
Pathology	199	118	94.0 (87.5, 97.2)	75.8 (66.5, 83.1)	79.7 (71.5, 85.9)	92.6 (84.8, 96.6)
Imaging	200	94	89.9 (82.4, 94.4)	95.0 (88.9, 97.9)	94.7 (88.1, 97.7)	90.6 (83.5, 94.8)
Future test data						
Pathology	200	129	93.0 (86.3, 96.6)	64.0 (54.2, 72.7)	72.1 (63.8, 79.1)	90.1 (81.0, 95.1)
Imaging	200	85	78.0 (68.9, 85.0)	93.0 (86.3, 96.6)	91.8 (84.0, 96.0)	80.9 (72.7, 87.0)

* ‘excl gene test’: for pathology reports, sensitivity is also reported when excluding supplementary reports that describe gene testing without providing explicit keywords for CRC. Confidence intervals are 95% Wilson intervals.

CRC, colorectal cancer.

Reductions in sensitivity and NPV on test data pathology reports were primarily due to supplementary reports that described genetic testing for CRC (online supplemental Table S6): these tests were done only when CRC was confirmed, but keywords for mismatch repair and other gene tests had not been included in the algorithm. When these 44 supplementary reports were ignored, the sensitivity on future OUH pathology reports increased to 96.5% and NPV to 95.5%, PPV dropped to 92.1% and specificity remained 90.1%. Similarly, the sensitivity on training data pathology reports would have increased to 98.9% and NPV to 98.7%, PPV would have dropped to 93.6% and specificity would have remained 92.6% if the 29 supplementary gene testing reports would have been excluded. It is relevant to also report the performance without supplementary gene testing reports, because identification of the main CRC reports is the primary use case. The drop in PPV on future imaging reports was due to 22 reports misclassified for various reasons. See online supplemental table S6 for enumeration of errors.

### TNM staging: T, N and M categories

The performance is first reported by considering the non-retrieval of implicit stages and the retrieval of historical stages as errors. In pathology reports, PPVs for the T, N and M categories were 98.0% (T), 98.9% (N) and 98.6% (M) in training data; and 94.0% (T), 100.0% (N) and 100.0% (M) in test data. Sensitivities for the T, N and M categories in pathology reports were 63.6% (T), 98.9% (N) and 95.9% (M) in training data; and 70.7% (T), 100.0% (N) and 100% (M) in test data. The corresponding NPVs were 45.0% (T), 99.1% (N) and 97.7% (M) in training data; and 67.0% (T), 100.0% (N) and 100.0% (M) in test data. The corresponding specificities were 97.8% (T), 99.1% (N) and 99.2% (M) in training data; and 95.7% (T), 100.0% (N) and 100.0% (M) in test data (table 4).

**Table 4:** Performance of a TNM stage algorithm for detecting the maximum value of each TNM category in the clinical reports of patients with CRC in OUH

TNM category	Num report	Num value	PPV (micro)	NPV	Sensitivity (micro)	Specificity
Pathology reports (subset of training data)						
T_pre	200	145	99.0 (94.5, 99.8)	53.5 (43.8, 62.9)	67.6 (59.6, 74.7)	98.2 (90.4, 99.7)
T	200	154	98.0 (93.0, 99.4)	45.0 (35.6, 54.8)	63.6 (55.8, 70.8)	97.8 (88.7, 99.6)
N	200	88	98.9 (93.8, 99.8)	99.1 (95.1, 99.8)	98.9 (93.8, 99.8)	99.1 (95.1, 99.8)
M	200	74	98.6 (92.5, 99.8)	97.7 (93.3, 99.2)	95.9 (88.7, 98.6)	99.2 (95.6, 99.9)
V	200	91	100.0 (95.9, 100.0)	100.0 (96.6, 100.0)	100.0 (95.9, 100.0)	100.0 (96.6, 100.0)
L	200	91	100.0 (95.9, 100.0)	100.0 (96.6, 100.0)	100.0 (95.9, 100.0)	100.0 (96.6, 100.0)
Pn	200	74	100.0 (94.9, 100.0)	98.4 (94.5, 99.6)	97.3 (90.7, 99.3)	100.0 (97.0, 100.0)
R	200	91	98.9 (94.0, 99.8)	100.0 (96.6, 100.0)	98.9 (94.0, 99.8)	100.0 (96.6, 100.0)
SM	200	7	87.5 (52.9, 97.8)	100.0 (98.0, 100.0)	100.0 (64.6, 100.0)	99.5 (97.1, 99.9)
H	200	0	–	100.0 (98.1, 100.0)	–	100.0 (98.1, 100.0)
G	200	15	100.0 (34.2, 100.0)	93.4 (89.1, 96.1)	13.3 (3.7, 37.9)	100.0 (98.0, 100.0)
Imaging reports (subset of training data)						
T_pre	200	42	100.0 (51.0, 100.0)	80.6 (74.5, 85.5)	9.5 (3.8, 22.1)	100.0 (97.6, 100.0)
T	200	128	96.0 (90.2, 98.4)	72.0 (62.5, 79.9)	75.0 (66.8, 81.7)	100.0 (94.9, 100.0)
N	200	96	97.7 (92.1, 99.4)	91.1 (84.3, 95.1)	89.6 (81.9, 94.2)	98.1 (93.3, 99.5)
M	200	54	97.2 (85.8, 99.5)	88.4 (82.6, 92.5)	64.8 (51.5, 76.2)	99.3 (96.2, 99.9)
V	200	27	100.0 (87.5, 100.0)	100.0 (97.8, 100.0)	100.0 (87.5, 100.0)	100.0 (97.8, 100.0)
Future pathology reports (test data)						
T_pre	200	133	100.0 (96.3, 100.0)	66.3 (56.7, 74.8)	74.4 (66.4, 81.1)	100.0 (94.6, 100.0)
T	200	130	94.0 (87.5, 97.2)	67.0 (57.3, 75.4)	72.3 (64.1, 79.3)	95.7 (88.1, 98.5)
N	200	79	100.0 (95.4, 100.0)	100.0 (96.9, 100.0)	100.0 (95.4, 100.0)	100.0 (96.9, 100.0)
M	200	8	100.0 (67.6, 100.0)	100.0 (98.0, 100.0)	100.0 (67.6, 100.0)	100.0 (98.0, 100.0)
V	200	89	100.0 (95.9, 100.0)	100.0 (96.7, 100.0)	100.0 (95.9, 100.0)	100.0 (96.7, 100.0)
L	200	90	100.0 (95.8, 100.0)	97.3 (92.5, 99.1)	96.7 (90.7, 98.9)	100.0 (96.6, 100.0)
Pn	200	77	100.0 (93.8, 100.0)	86.6 (80.0, 91.3)	75.3 (64.6, 83.6)	100.0 (97.0, 100.0)
R	200	90	100.0 (95.9, 100.0)	100.0 (96.6, 100.0)	100.0 (95.9, 100.0)	100.0 (96.6, 100.0)
SM	200	4	100.0 (51.0, 100.0)	100.0 (98.1, 100.0)	100.0 (51.0, 100.0)	100.0 (98.1, 100.0)
H	200	1	100.0 (20.7, 100.0)	100.0 (98.1, 100.0)	100.0 (20.7, 100.0)	100.0 (98.1, 100.0)
G	200	84	100.0 (56.6, 100.0)	59.5 (52.5, 66.1)	6.0 (2.6, 13.2)	100.0 (96.8, 100.0)
Future imaging reports (test data)						
T_pre	200	132	50.0 (15.0, 85.0)	34.7 (28.4, 41.6)	1.5 (0.4, 5.4)	100.0 (94.7, 100.0)
T	200	138	93.9 (87.4, 97.2)	61.4 (51.6, 70.3)	67.4 (59.2, 74.6)	100.0 (94.2, 100.0)
N	200	105	99.0 (94.3, 99.8)	91.3 (84.4, 95.4)	90.5 (83.4, 94.7)	100.0 (96.1, 100.0)
M	200	89	100.0 (95.4, 100.0)	92.5 (86.4, 96.0)	89.9 (81.9, 94.6)	100.0 (96.7, 100.0)
V	200	35	100.0 (89.6, 100.0)	98.8 (95.7, 99.7)	94.3 (81.4, 98.4)	100.0 (97.7, 100.0)

‘Num value’ is the number of reports that contains information about a TNM category (eg, a T staging value for the T category). PPV (micro) is the micro-average of positive predictive values for detecting each TNM value when it was present; NPV is the negative predictive value for correctly detecting that no TNM values were present; sensitivity (micro) is the micro-average of sensitivities for detecting each TNM value when it was present. 95% Wilson confidence intervals are shown in brackets.

CRC, colorectal cancer; NPV, negative predictive value; PPV, positive predictive value.

In imaging reports, the T, N and M categories had PPVs of 96.0% (T), 97.7% (N) and 97.2% (M) in training data; and 93.9% (T), 99.0% (N) and 100.0% (M) in test data. The sensitivities for the T, N and M categories in imaging reports were 75.0% (T), 89.6% (N) and 64.8% (M) in training data; and 67.4% (T), 90.5% (N), and 89.9% (M) in test data. The corresponding NPVs were 72.0% (T), 91.1% (N) and 88.4% (M) in training data; and 61.4% (T), 91.3% (N) and 92.5% (M) in test data. The corresponding specificities were 100.0% (T), 98.1% (N) and 99.3% (M) in training data; and 100.0% (T), 100.0% (N) and 100.0% (M) in test data (table 4).

When T scores were not detected, it was mainly due to reports where the absence of tumour could be inferred or where the tumour could not be assessed, but where T stage was not explicitly reported as T0 or TX. Similarly, when M scores were not correctly detected in imaging reports, it was often when metastasis was absent or not assessed but was not explicitly reported as M0 or MX (implicit M staging). The remaining errors were due to misclassifying historic staging as present staging (16 errors), misclassifying some MRI sequences as T-stages (two errors) and other reasons. See online supplemental materials S7 for a complete enumeration of errors. A simplified confusion matrix for the detection of T, N and M scores is given in online supplemental table S8.

For the task of extracting TNM scores given in letters and numbers, historical or current, the algorithm made no errors over the 400 pathology reports, and made six errors over the 400 imaging reports (online supplemental table S7).

### TNM staging: other categories

The venous (V) and lymphatic (L) invasion scores were detected with 100% PPV and at least 94.3% sensitivity in both pathology and imaging reports (table 4). The residual tumour status (R) and perineural invasion (Pn) were detected with at least 98.9% PPV and 75.3% sensitivity in pathology reports (table 4). There were too few examples of Kikuchi (11) and Haggit scores (1). Tumour regression grade (G) had low sensitivity (less than 14%) as it was usually not reported with letter ‘G’.

### Running time

The CRC algorithm ran for 6.6 min and the TNM algorithm for 34.58 min on 10 000 pathology and 10 000 imaging reports on a computer with Intel Xeon Platinum 8370C CPU (2.80 GHz) and 64 GB RAM (single core). The code repository also contains a function to run it on parallel cores to make it faster.

## Discussion

Two algorithms were developed to extract primary CRC status (present/absent) and TNM staging scores from imaging and histopathology reports, facilitating clinical research using electronic health records. The CRC algorithm achieved at least 92% PPV and 72% sensitivity in pathology reports and 78% PPV and 92% sensitivity in imaging reports. Reduced sensitivity in pathology was mainly due to supplementary gene testing reports that may not need to be detected, and reduced PPV in imaging reports was due to greater language variations. The TNM algorithm achieved at least 94% PPV and 64% sensitivity across all T/N/M categories and report types. Reduced sensitivity was mainly due to non-retrieval of implicit staging. For extracting explicit stages, historical or current, the TNM algorithm made no errors over 400 pathology reports and six errors over 400 imaging reports.

### Potential usage

The CRC detection component helps identify clinical reports that discuss current primary CRC, which can be used for extracting the TNM stage, for deriving a timestamped outcome variable for research studies (eg, the date of the first pathology report that discusses primary CRC can serve as the date of cancer), as part of a cohort definition for a research project, and for checking the concordance of different cancer data sources. If a project requires high precision, the outputs may need additional review. The algorithm facilitates this by outputting extracted tumour keywords with surrounding text. Even though the CRC algorithm was developed on reports of patients with CRC (some of which describe other tumours and non-primary tumours), it can likely identify primary CRC reports within a broader population, because it was designed to exclude non-primary CRC or history of CRC.

The TNM extraction algorithm can help describe the severity of CRC and allows the selection of patients with a similar disease profile for analyses such as comparative treatment effectiveness studies. It could also help with data quality checks—TNM stages reported in histopathology reports can be checked against staging in other databases. However, the usefulness of the TNM staging algorithm depends on two factors: (1) whether the reports usually give TNM staging in letters and numbers (the algorithm cannot infer TNM staging), and (2) whether the reports usually give more than one TNM staging value in a sequence (eg, ‘T0 N0’) as the performance of disambiguating single TNM values from false positives is less reliable.

The TNM algorithm differs from CanStaging+,^20^ a staging tool used by cancer registries: the algorithm automatically extracts staging from free text if it is given in letters and numbers, whereas CanStaging+ requires a user to input information about the tumour to receive a staging score.

Application of the algorithms requires basic familiarity with the python programming language. The associated code repository provides examples.

### Related work

Several attempts have been made to extract explicitly given TNM staging values from free text.^3^,^12^ It is hard to evaluate how thorough and flexible these methods are, as only two studies have publicly available source code^6 7^ and one provides extraction rules in their publication.^12^ The available source codes^21 22^ and rules are less generalisable than the current algorithm (online supplemental materials S9).

### Strengths and limitations

The algorithms were iteratively developed in collaboration with CRC clinicians on more than 58 000 reports, predominantly from Oxford University Hospitals and Royal Marsden NHS FTs, helping ensure they cover variations in reporting. The algorithms rely on three core python packages and run for roughly half an hour on a single core per 20 000 reports, which should make them relatively easy to deploy in hospital research and information systems. The algorithms also save all extracted tumour keywords and TNM staging phrases with left and right context, which can be semi-manually reviewed to increase precision and sensitivity and provide assurance. The TNM extraction algorithm is likely to generalise well to other datasets and cancers, as TNM staging is reported in specific ways using letters and numbers.

The algorithms do not apply spell checking, so if clinical reports contain spelling errors for tumour keywords and anatomical sites, then the CRC detection algorithm may not work well. The CRC detection algorithm also relies on manually curated patterns that may not generalise equally well to reports from other centres. The TNM algorithm extracts only stages reported in letters and numbers and cannot infer the staging if sufficient information is nevertheless given in the report. The TNM algorithm does not extract the TNM coding edition. Finally, the algorithms were developed on a multicentre dataset but evaluated only on OUH data due to resource and access constraints.

### Potential machine learning approaches

The CRC status and TNM scores could have been extracted using a large language model (LLM) or by fine-tuning a bidirectional transformer-encoder (BERT) machine learning (ML) model^23 24^ to classify text extracts. Due to more nuanced processing of context, these models could more accurately identify CRC status, distinguish historical and current TNM stages and infer implicit staging. However, ML models would unlikely be beneficial over the current algorithm for explicit staging, given the limited formats of TNM scores. ML models would also have higher computational cost, and LLMs would have potential issues with data privacy and lack of confidence scores for predictions (online supplemental Materials S10).

### Future directions

The CRC algorithm can be combined with an ML model to increase its precision (online supplemental Materials S10). The TNM algorithm can be supplemented by ML models as noted above, especially for inferring implicit staging. Given variability in report texts, the development of regex patterns for inferring implicit staging may be more effortful than fine-tuning an ML model. Ideally, the current information extraction pipeline would also be expanded to cover the Royal College of Pathologists’ minimum dataset requirements.^25^

## Conclusion

The CRC and TNM algorithms were accurate on OUH data, but outputs may need semi-manual review if used in other medical centres or if higher precision is required. The CRC algorithm will likely remain useful when a lightweight tool is needed or as a comparator against more complex models. The TNM algorithm is expected to remain useful for extracting the TNM scores given in letters-numbers but would need to be supplemented by ML models for inferring implicit staging.

## Supplementary material

10.1136/bmjhci-2025-101521 online supplemental file 1

## Data Availability

No data are available.
