# Supplementary material for: Supporting cancer research on real-world data: extracting colorectal cancer status and explicitly written TNM stages from free-text imaging and histopathology reports
Source: BMJ Health Care Inform. 2025 Sep 21;32(1):e101521. doi: 10.1136/bmjhci-2025-101521 (PMC12458752; doi:10.1136/bmjhci-2025-101521)
Supplement: online supplemental file 1 [file bmjhci-32-1-s001.pdf]

Supplementary Materials for “Supporting cancer research on real-world data: Extracting colorectal cancer status and explicitly written TNM stages from free-text imaging and histopathology reports”

24 July 2025

Table of contents

S1. TNM classification for colorectal tumours 2
S2. Tripod-LLM checklist 3
S3. The colorectal cancer detection algorithm 8
S4. The TNM stage extraction algorithm 12
S5. Evaluation of the CRC and TNM extraction algorithms 13
  Selection of clinical reports 13
  Annotation of selected reports 14
  Performance metrics 15
S6. Errors of the CRC detection algorithm 16
S7. Errors of the TNM stage extraction algorithm 17
S8. Simplified confusion matrix for the TNM staging algorithm 18
S9. Other TNM stage extraction algorithms 20
S10. Potential machine learning approaches 22

S1. TNM classification for colorectal tumours

The main categories of the TNM pathological classification system for colorectal tumours are given in Table S1, according to the Union for International Cancer Control (UICC) TNM 8th edition [1]. These were taken from the freely published version included in the "Dataset for histopathological reporting of colorectal cancer" by the Royal College of Pathologists[2]. A simplified description is also available on the Cancer Research UK website (https://www.cancerresearchuk.org/about-cancer/bowel-cancer/stages-types-and-grades/TNM-staging).

Table S1. Union for International Cancer Control TNM 8 pathological classification of colorectal tumours

Example	Comment
**pT (primary tumour)**	
pTX	Primary tumour cannot be assessed.
pT0	No evidence of primary tumour.
pT1	Tumour invades submucosa.
pT2	Tumour invades muscularis propria.
pT3	Tumour invades into subserosa or into non-peritonealised pericolic or perirectal tissues.
pT4	
pT4a	Tumour perforates visceral peritoneum.
pT4b	Tumour directly invades other organs or structures.
**N (regional lymph nodes)**	
pNX	Regional lymph nodes cannot be assessed.
pN0	No regional lymph node metastatic disease.
pN1	Metastatic disease in 1–3 regional lymph nodes.
pN1a	Metastasis in 1 regional lymph node.
pN1b	Metastases in 2–3 regional lymph nodes.
pN1c	Tumour deposit(s), i.e. satellites, in the subserosa, or in non-peritonealised pericolic or perirectal soft tissue without regional lymph node metastatic disease (tumour deposits are ignored if there is nodal metastatic disease).
pN2	Metastatic disease in 4 or more regional lymph nodes.
pN2a	Metastases in 4–6 regional lymph nodes.
pN2b	Metastases in 7 or more regional lymph nodes.
**M (distant metastasis)**	
pM0	Cancer has not spread to other organs.
pM1	Distant metastatic disease.
pM1a	Metastasis confined to 1 organ without peritoneal metastases.
pM1b	Metastases in more than 1 organ.
pM1c	Metastases to the peritoneum with or without other organ involvement.

S2. Tripod-LLM checklist

This study is reported according to the Tripod-LLM checklist where applicable; items relevant to large-language models were left out. The checklist can be found below (page numbers refer to the submitted manuscript).

This checklist is filled in for the study "Supporting cancer research on real-world data: Extracting colorectal cancer status and explicitly written TNM stages from free-text imaging and histopathology reports". The study evaluates a regex-based tool, not an LLM, so LLM-specific items are marked as N/A.
—Andres Tamm (2025-03-31)

The TRIPOD-LLM Statement: A Targeted Guideline For Reporting Large Language Models Use

Supplementary Table 2: Fillable TRIPOD-LLM checklist

Section	Item	Checklist Item	Research Design	LLM Task	Page
Title					
Title	1	Identify the study as developing, fine-tuning, and/or evaluating the performance of an LLM, specifying the task, the target population, and the outcome to be predicted.	All	All	1
Abstract					
Abstract	2	See TRIPOD-LLM for Abstracts	All	All	2
Introduction					
Background	3a	Explain the healthcare context / use case (e.g., administrative, diagnostic, therapeutic, clinical workflow) and rationale for developing or evaluating the LLM, including references to existing approaches and models.	All	All	3
	3b	Describe the target population and the intended use of the LLM in the context of the care pathway, including its intended users in current gold standard practices (e.g., healthcare professionals, patients, public, or administrators).	E<br>H	All	3,9
Objectives	4	Specify the study objectives, including whether the study describes the initial development, fine-tuning, or validation of an LLM (or multiple stages).	All	All	3
Methods					
Data	5a	Describe the sources of data separately for the training, tuning, and/or evaluation datasets and the rationale for using these data (e.g., web corpora, clinical research/trial data, EHR data, or unknown).	All	All	4,5
	5b	Describe the relevant data points and provide a quantitative and qualitative description of their distribution and other relevant descriptors of the dataset (e.g., source, languages, countries of origin)	All	All	4
	5c	Specifically state the date of the oldest and newest item of text used in the development process (training, fine-tuning, reward modeling) and in the evaluation datasets.	All	All	4,5
	5d	Describe any data pre-processing and quality checking, including whether this was similar across text corpora, institutions, and relevant socio-demographic groups.	All	All	4
	5e	Describe how missing and imbalanced data were handled and provide reasons for omitting any data.	All	All	5
Analytical Methods	6a	Report the LLM name, version, and last date of training.	All	All	N/A (no LLM)
	6b	Report details of LLM development process, such as LLM architecture, training, fine-tuning procedures, and alignment	M<br>D	All	4

		strategy (e.g., reinforcement learning, direct preference optimization, etc.) and alignment goals (e.g., helpfulness, honesty, harmlessness, etc.).			
	6c	Report details of how text was generated using the LLM, including any prompt engineering (including consistency of outputs), and inference settings (e.g., seed, temperature, max token length, penalties), as relevant.	M D E	All	N/A (no LLM)
	6d	Specify the initial and post-processed output of the LLM (e.g., probabilities, classification, unstructured text).	All	All	4,5, Supplement S2 and S3
	6e	Provide details and rationale for any classification and, if applicable, how the probabilities were determined and thresholds identified.	All	C OF	4,5, Supplement S2 and S3
LLM Output	7a	Include metrics that capture the quality of generative outputs, such as consistency, relevance, and accuracy, compared to gold standards.	All	QA IR DG SS MT	N/A (no LLM)
	7b	Report the outcome metrics' relevance to downstream task at deployment time and, where applicable, correlation of metric to human evaluation of the text for the intended use.	E H	All	5
	7c	Clearly define the outcome, how the LLM predictions were calculated (e.g., formula, code, object, API), the date of inference for closed-source LLMs, and evaluation metrics.	E H	All	4,5
	7d	If outcome assessment requires subjective interpretation, describe the qualifications of the assessors, any instructions provided, relevant information on demographics of the assessors, and inter-assessor agreement.	All	All	N/A (no LLM, no subjective outcomes)
	7e	Specify how performance was compared to other LLMs, humans, and other benchmarks or standards.	All	All	N/A (no LLM)
Annotation	8a	If annotation was done, report how text was labeled, including providing specific annotation guidelines with examples.	All	All	5, Supplement S4
	8b	If annotation was done, report how many annotators labeled the dataset(s), including the proportion of data in each dataset that were annotated by more than 1 annotator, and the inter-annotator agreement.	All	All	5, Supplement S4
	8c	If annotation was done, provide information on the background and experience of the annotators or characteristics of any models involved in labelling.	All	All	5, Supplement S4
Prompting	9a	If research involved prompting LLMs, provide details on the processes used during prompt design, curation, and selection.	All	All	N/A (no LLM)
	9b	If research involved prompting LLMs, report what data were used to develop the prompts.	All	All	N/A (no LLM)
Summarization	10	Describe any preprocessing of the data before summarization.	All	SS	4
Instruction tuning/Alig	11	If instruction tuning/alignment strategies were used, what were the instructions, data, and interface used for evaluation,	M D	All	N/A (no LLM)

nment		and what were the characteristics of the populations doing evaluation?			
Compute	12	Report compute, or proxies thereof (e.g., time on what and how many machines, cost on what and how many machines, inference time, floating-point operations per second (FLOPs)), required to carry out methods.	M D E	All	9
Ethical Approval	13	Name the institutional research board or ethics committee that approved the study and describe the participant-informed consent or the ethics committee waiver of informed consent.	All	All	11
Open Science	14a	Give the source of funding and the role of the funders for the present study.	All	All	11
	14b	Declare any conflicts of interest and financial disclosures for all authors.	All	All	11
	14c	Indicate where the study protocol can be accessed or state that a protocol was not prepared.	H	All	No protocol
	14d	Provide registration information for the study, including register name and registration number, or state that the study was not registered.	H	All	Not registered
	14e	Provide details of the availability of the study data.	All	All	11
	14f	Provide details of the availability of the code to reproduce the study results.	All	All	11
Public Involvement	15	Provide details of any patient and public involvement during the design, conduct, reporting, interpretation, or dissemination of the study or state no involvement.	H	All	No involve-ment
Results					
Participants	16a	When using patient/EHR data, describe the flow of text/EHR/patient data through the study, including the number of documents/questions/participants with and without the outcome/label and follow-up time as applicable.	E H	All	7,8 (Tables 3 and 4)
	16b	When using patient/EHR data, report the characteristics overall and, for each data source or setting, and for development/evaluation splits, including the key dates, key characteristics, and sample size.	E H	All	N/A (demo-graphics not essential for the analysis)
	16c	For LLM evaluation that include clinical outcomes, show a comparison of the distribution of important clinical variables that may be associated with the outcome between development and evaluation data, if available.	E H	All	N/A (no other essential variables to report)
	16d	When using patient/EHR data, specify the number of participants and outcome events in each analysis (e.g., for LLM development, hyperparameter tuning, LLM evaluation).	E H	All	7,8 (Tables 3 and 4)
Performance	17	Report LLM performance according to pre-specified metrics (see item 7a) and/or human evaluation (see item 7d).	All	All	7,8 (Tables 3 and 4)
LLM Updating	18	If applicable, report the results from any LLM updating, including the updated LLM and subsequent performance.	All	All	N/A (no LLM)

Discussion					
Interpretation	19a	Give an overall interpretation of the main results, including issues of fairness in the context of the objectives and previous studies.	All	All	9
Limitations	19b	Discuss any limitations of the study and their effects on any biases, statistical uncertainty, and generalizability.	All	All	10
Usability of the LLM in context	19c	Describe any known challenges in using data for the specified task and domain context with reference to representation, missingness, harmonization, and bias.	E H	All	3
	19d	Define the intended use for the implementation under evaluation, including the intended input, end-user, level of autonomy/human oversight.	E H	All	9
	19e	If applicable, describe how poor quality or unavailable input data should be assessed and handled when implementing the LLM, i.e., what is the usability of the LLM in the context of current clinical care.	E H	All	10
	19f	If applicable, specify whether users will be required to interact in the handling of the input data or use of the LLM, and what level of expertise is required of users.	E H	All	9
	19g	Discuss any next steps for future research, with a specific view to applicability and generalizability of the LLM.	All	All	10

LLM = large language model; M = LLM methods; D = *de novo* LLM development; E = LLM evaluation; H = LLM evaluation in healthcare settings; C = classification; OF = outcome forecasting; QA = long-form question-answering; IR = information retrieval; DG = document generation; SS = summarization and simplification; MT = machine translation; EHR = electronic health record.

Note: For studies using existing LLMs, users should include reference(s) to reportable information if provided by the original developers or state that this information is not available.

S3. The colorectal cancer detection algorithm

To detect whether a clinical report discusses current primary colorectal cancer (CRC), it was processed three stages:

1. Identify keywords referring to colorectal tumours, including direct references such as “colorectal cancer” and general tumour keywords preceded or followed by a relevant anatomical site.
2. Exclude tumour keywords that have one of the following statuses: negated, general, historic, possible (i.e. not affirmative), metastatic, or treatment response (see Table 3 in main text).
3. Decide that a clinical report mentions current CRC, if it has at least one tumour keyword that was not marked for exclusion in the previous step.

We used an extensive vocabulary of keywords for tumours and sites created with input from ND and HJ (Table S3a). In Table S3a, the tumour keywords are given in a simplified form and are later translated into more flexible regular expressions.

In Step 2, the statuses of tumour keywords were identified using a ConText-like algorithm [15]: we looked for certain keywords on the left and right sides of the tumour keywords (such as "negative" for the "negated" status, see Table S3b and S3c for examples), and accepted these only if certain termination keywords did not occur between the status keyword and tumour keywords (such as "although" and "apart"). Diverging from the original ConText algorithm, the same termination keywords were not used for all statuses; the status keywords were considered only if they occurred within a maximum allowed distance from the tumour keywords; and pseudo-trigger terms were not applied – this seemed to yield better results during iterative algorithm development. Some keywords for detecting the assertion statuses were taken from the python implementation of ConText (https://github.com/chapmanbe/pyConTextNLP).

Table S3a. Patterns for detecting colorectal tumours and tumour sites

Pattern	Concept	Concept ID	Pattern type
(ca?ecumlca?ecal)	caecum	1	wordstart
right (colonlhemicolon)	right (ascending) colon	2	wordstart
ascending colon	right (ascending) colon	2	wordstart
right hemicolect	right (ascending) colon	2	wordstart
hepatic flex	hepatic flexure	3	wordstart
right colic flex	hepatic flexure	3	wordstart
transverse colon	transverse colon	4	wordstart
splenic flex	splenic flexure	5	wordstart
left colic flex	splenic flexure	5	wordstart
left (colonlhemicolon)	left (descending) colon	6	wordstart
descending colon	left (descending) colon	6	wordstart
left hemicolect	left (descending) colon	6	wordstart
sigmoid	sigmoid colon	7	wordstart
(ano)?(rectumlrectal)	rectum	8	wordstart
anallanus	anus	35	word
transanal	rectum	8	wordstart
anorectal	rectum	8	wordstart
colon	colon	9	wordend
(coloniclcolonos)	colon	9	string
colectomy	colon	9	string
colo?-?rect	colon and rectum	10	string
crc	colon and rectum	10	word
dukes	colon and rectum	10	word
large (bowellintestine)	colon and rectum	10	word
bowel wall	colon and rectum	10	word
rectosigmoid	colon and rectum	10	word

<table>
<tr><td>recto(-|/)?sigmoid</td><td>colon and rectum</td><td>10</td><td>word</td></tr>
<tr><td>kikuchi|hagg?itt?</td><td>colon and rectum</td><td>10</td><td>word</td></tr>
<tr><td>(tumour|tumor|carcinom|cancer|carcinoid)</td><td>tumour</td><td>11</td><td>string</td></tr>
<tr><td>malignant (\w+){,2}neoplas</td><td>tumour</td><td>11</td><td>wordstart</td></tr>
<tr><td>crc</td><td>crc</td><td>12</td><td>word</td></tr>
<tr><td>colo-?rectal (tumour|tumor|carcinom|neoplas|cancer)</td><td>crc</td><td>12</td><td>string</td></tr>
<tr><td>liver|segments? ([1-8]|i{1,3}|iv|v|vi{1,3})[ab]?</td><td>liver</td><td>15</td><td>word</td></tr>
<tr><td>hepatic (?!flex\w*)</td><td>liver</td><td>15</td><td>word</td></tr>
<tr><td>hepatectom</td><td>liver</td><td>15</td><td>wordstart</td></tr>
<tr><td>pulmonar</td><td>lung</td><td>16</td><td>wordstart</td></tr>
<tr><td>lung|upper lobe|middle lobe|lower lobe</td><td>lung</td><td>16</td><td>wordstart</td></tr>
<tr><td>peritoneum|peritoneal</td><td>peritoneum</td><td>17</td><td>word</td></tr>
<tr><td>omentum|omental</td><td>omentum</td><td>18</td><td>wordstart</td></tr>
<tr><td>pelvis|pelvic</td><td>pelvis</td><td>19</td><td>word</td></tr>
<tr><td>uterus|uterin|myometri|endometri</td><td>uterus</td><td>20</td><td>wordstart</td></tr>
<tr><td>ovary|ovari</td><td>ovaries</td><td>21</td><td>wordstart</td></tr>
<tr><td>bladder|urinar|ureter</td><td>bladder</td><td>22</td><td>wordstart</td></tr>
<tr><td>small (bowel|intestine)</td><td>small intestine</td><td>23</td><td>word</td></tr>
<tr><td>duoden|jejun|ileum|ileal|ileo</td><td>small intestine</td><td>23</td><td>wordstart</td></tr>
<tr><td>mesentery|mesenteric</td><td>mesentery</td><td>24</td><td>wordstart</td></tr>
<tr><td>spleen</td><td>spleen</td><td>25</td><td>wordstart</td></tr>
<tr><td>anastomo</td><td>anastomosis</td><td>26</td><td>wordstart</td></tr>
<tr><td>adrenal</td><td>adrenal gland</td><td>27</td><td>wordstart</td></tr>
<tr><td>kidney|renal</td><td>kidney</td><td>28</td><td>wordstart</td></tr>
<tr><td>abdomen|abdominal|belly</td><td>abdomen</td><td>29</td><td>word</td></tr>
<tr><td>node|noda</td><td>nodes</td><td>30</td><td>wordstart</td></tr>
<tr><td>bone|bony|marrow</td><td>bone</td><td>31</td><td>wordstart</td></tr>
<tr><td>pleura</td><td>pleura</td><td>32</td><td>wordstart</td></tr>
<tr><td>brain|mening</td><td>brain</td><td>33</td><td>wordstart</td></tr>
<tr><td>(temporal|occipital|frontal|parietal) lobe</td><td>brain</td><td>33</td><td>wordstart</td></tr>
<tr><td>head|face|neck|tongue</td><td>head</td><td>34</td><td>word</td></tr>
<tr><td>abdomino-?perineal resect|abdominal perineal resect|anterior resect</td><td>rectum</td><td>8</td><td>wordstart</td></tr>
<tr><td>gallbladder</td><td>gallbladder</td><td>36</td><td>wordstart</td></tr>
<tr><td>retroperitoneum|retroperitoneal</td><td>retroperitoneum</td><td>37</td><td>wordstart</td></tr>
<tr><td>cervix|cervical</td><td>cervix</td><td>38</td><td>word</td></tr>
<tr><td>mesorectum|mesorectal</td><td>mesorectum</td><td>39</td><td>word</td></tr>
<tr><td>(bile|biliary|hepatic|cystic|gall|hepatopancreatic) duct</td><td>bile duct</td><td>40</td><td>word</td></tr>
<tr><td>choledochus</td><td>bile duct</td><td>40</td><td>word</td></tr>
<tr><td>prostat</td><td>prostate</td><td>41</td><td>wordstart</td></tr>
</table>

Note. Each pattern is additionally processed: if it is ‘wordstart’, then start-of-word markers are added in front; if it is ‘wordend’ then end-of-word markers are added in the end; if it is ‘word’ then both start-of-word and end-of-word markers are added. Furthermore, gaps between words in the pattern are made flexible such that they can contain a certain number of characters and other words.

Table S3b. Simplified examples of assertion statuses assigned to colorectal tumour keywords

Assertion status	Example
Negated	‘no evidence of colorectal cancer’
General	‘in patients with colorectal cancer’
Historic	‘history of colorectal cancer’
Possible	‘suspicious for colorectal cancer’
Metastatic or recurrent	‘recurrent colorectal cancer’
Treatment response	‘reduction in size of colorectal tumour’

Table S3c. Patterns that help identify when cancer is not discussed in the present and definite sense

Category	Pattern	Pattern type	Side	Termination patterns (left)	Termination patterns (right)	Distance
negated	: (no|none|negative)	word	right	stop|stop4		4
negated	(absent|excluded|free|negative|resolved|ruled out)	word	right	stop|stop2|stop4		40
negated	not (demonstrated|identified|indicated|known|present|seen|significant|suggested)	word	right	stop		40
negated	cannot be seen	word	right	stop		40
negated	(clear|free) (of|from)	word	left	stop2	stop	100
negated	(nil|no|not|without)	word	left		stop|stop3	100
negated	no (evidence|features|indication|sign)	wordstart	left		stop	40
negated	not (contain|indicat|represent|show|suggest)	wordstart	left		stop	40
negated	cannot see	word	left		stop	40
negated	absence of	word	left	stop2	stop	40
negated	(is|are) negative for	word	left		stop	40
negated	preferred over	word	left		stop	40
historic	(histor|previous|predates|prior|known|recent|has had)	wordstart	left		stoph|stoph2	40
historic	clinical (details|history|information)	wordstart	left		stoph	80
historic	(19\d\d|20\d\d)	word	left		stoph|stopr	40
historic	(jan|feb|mar|apr|in may|may 19\d\d|may 20\d\d|jun|jul|aug|sep|oct|nov|dec)	word	left		stoph|stopr	40
historic	(january|february|march|april|in may|may 19\d\d|may 20\d\d|june|july|august|september|october|november|december)	word	left		stoph|stopr	40
historic	(19\d\d|20\d\d)	word	right	stoph|stopr		20
historic	(jan|feb|mar|apr|may 19\d\d|in may|may 20\d\d|jun|jul|aug|sep|oct|nov|dec)	word	right	stoph|stopr		20
historic	(january|february|march|april|in may|may 19\d\d|may 20\d\d|june|july|august|september|october|november|december)	word	right	stoph|stopr		20
general	(individuals|patients|subjects) with	word	both			40
general	(not) (typical) (for)	word	left		stop	40
general	(correlat\w*)	word	left		stop	40
general	(lesions|tumours) (can|may|might|could) be associated with	word	left		stop	40
general	(can|may|might|could) be associated with	word	right	stop		20
general	(genome|genomes|genes|resistance|mutation|mutations|antibody|antibodies)	word	both			40
general	(recommend\w*)	word	left		stop	40
general	in (colorectal cancer|crc|tumours|tumors)	word	right			40
general	can show	word	right	stop		20
general	in the context of	word	left		stop	40
general	(heterogeneity|heterogeneous)	word	right	stop		10
general	clinical trials? of	word	left		stop	80
general	to (exclude|rule out)	wordstart	left		stop	80
general	tumou?r types	wordstart	both			40
general	(guidelines|guidance|guide|evaluation of)	wordstart	both			40
general	(origin\w*|source\w*|derive\w*) (of|from)	word	left		stop	40
general	(options|therapies|therapy|treatments|treatment) (in|for)	word	left		stop	40
general	(investigation|investigating|investigate|test|tests|testing|study|studies|research|res)	word	left		stop	40
general	(investigation|investigating|investigate|test|tests|testing|study|studies|research|res)	word	right	stop		40
general	(pathway|clinical)	word	left		stop	10
general	(pathway)	word	right	stop		10
general	(colonoscopy|colonoscopies) (advis\w*|recommend\w*|suggest\w*)	string	left			100
general	(variant|variants|variation|variations)	word	both			40
general	panel	word	right			40
general2	(occurring|occurs|reported|frequency|frequencies|frequently|gene) in	word	left			10
general2	in this	word	left			10
metastatic	(metastatic|recurrent|recurring)	word	both			40
response	(response|respond|responds|regression|trg|trs)	word	right			10

response	(reduction) in	word	left		stop	10
response	(residuallremaining)	word	left		stop	10
possible	(suspectedlpresumedlsuggestedlprobablellikelylpossiblelnot definite)	wordstart	right	stopr		40
possible	(mightlmaylcouldlcan) be (consideredlpresentlassociated)	wordstart	right	stopr		20
possible	(should belis) (consideredlpossiblela? possibilitylpossibilities)	wordstart	right	stopr		20
possible	(cannot be) (ruled outlexcluded)	wordstart	right	stopr		40
possible	(difficultlnot possiblelimpossiblelhard) to (rule outlexclude)	wordstart	right	stopr		40
possible	(suspiciouslconcerning) for	word	left		stopr	40
possible	presumelsuggestlsuspectlsuspiclprobabllikelpossiblnot definit	wordstart	left		stopr	40
possible	(appears tolmightlcouldlmaylunlikely to) (representlindicatelshowlbelreflect)	word	left		stopr	40
possible	(appears tolmightlcouldlmaylunlikely to) be (associated withlrelated to)	word	left		stopr	40
possible	(shouldlmightlcouldlmay) be considered as	word	left		stopr	40
possible	may or may not	word	left		stopr	40
possible	(if it islif this is)	word	left		stopr	40
possible	(if)	word	left		stopr	5
possible	or	word	left		stopr	5
possible	(cannotlunableldifficultlnot possiblelimpossiblelhardlinadequate) (to) (rule outlexclude)	word	left		stopr	40
possible	(importantlneededlnecessary) (to) (rule outlexclude)	word	left		stopr	40
possible	(preventslprecludes) (ruling outlexcluding)	word	left		stopr	40
stop	(althoughlapartlasidelbutlexceptliflhoweverlneverthelessforlfromlstilllthoughlyetlwhich)	word				
stop	(causeletiologyloriginlreasonlsource) (oflfor)	word				
stop2	(nolnotlcannotlwithout)	word				
stop3	(certainlchangldecreaslincreasldifficultylthreaten)	wordstart				
stop4	and	word				
stoph	present	wordstart				
stoph2	(haslhavelfor)	word				
stopr	(althoughlapartlasidelbutlexceptliflhoweverlneverthelesslthoughlyetlwithland)	word				

Note. Each pattern is additionally processed: if it is ‘wordstart’, the start-of-word markers are added in front; if it is ‘word’ the start-of-word and end-of-word markers are added. Furthermore, gaps between words in the pattern are made flexible such that they can contain a certain number of characters and other words. If the pattern is applied on the left (or right) side of the target keyword, certain other patterns can terminate it (“Termination patterns (left)”, “Termination patterns (right)”). The “Distance” indicates the maximum number of characters for how close the pattern must be to the target keyword to apply.

S4. The TNM stage extraction algorithm

TNM staging is commonly reported using a limited set of letters and numbers (Table 2), so it should be extractable by pattern matching with regular expressions. This was accomplished in four stages:

1. Extract phrases that contain TNM staging
    a. Extract phrases that contain a sequence of TNM values, such as "pT1 (text) N0 (3/10) M0 R0 V0 L0".
    b. Extract phrases that contain a single TNM value, such as "stage: pT1".
2. Filter and clean the phrases
    a. Split phrases that contain multiple T values at the T value. For example, "pT1 N0 M0 text pT2 N1 MX" would be split into "pT1 N0 M0 text" and "pT2 N1 MX".
    b. (optional) Identify historical phrases and mark these for exclusion.
    c. Identify unusual phrases such as multiple-choice prompts for T staging ("T 1 / 2 / 3 / 4"), and mark these for exclusion.
    d. Clean the phrases, retaining only TNM values. For example, "pT1 a & b (text) N0" would be replaced with "pT1a/1b N0".
3. Extract TNM values from the cleaned phrases. For example, if the cleaned phrase is “pT1a/1b N0”, then “1a” and “1b” will be the extracted T values and 0 will be N value. A total of 11 TNM categories were extracted, including T, N and M (Table 4). Values were only extracted from unique phrases to reduce running time.
4. For each clinical report, report the maximum and minimum values for each TNM category (as some reports may contain multiple TNM phrases).

Extracting TNM values in stages also allows for quality checks: the TNM phrases extracted at Step 1 can be examined along with their surrounding text.

The regular expressions that extract TNM phrases were designed to extract valid TNM phrases from anywhere in the report while avoiding false positives. Firstly, the building-block patterns that match individual TNM values were constrained, so that each TNM value must be preceded and/or followed by another TNM value (as in "t0n0m0") or be surrounded by nonword characters (as in "summary: t0 n0 m0."). Secondly, when extracting a sequence of TNM values, only certain common sequences were allowed, such as "T … N … M", "T … N … R", "T … N", among others, where "…" can contain text or other TNM values. Thirdly, when extracting TNM values that appear alone, a dictionary of keywords was used to retain or reject them. For example, "T1" was retained if preceded by the word "staging" (as in "staging: pT1") but rejected if closely followed by the word "no" (as in "tumour perforation (pT4): No"). On the other hand, the patterns were made flexible to avoid false negatives – they allowed for repetitions in TNM values, mis-spelling of 0 as O (only in sequences of values), gaps between letters and values, and comments between values.

During the cleaning stage, historical TNM-phrases were identified using a method similar to the ConText algorithm [15]. However, as most reports did not contain TNM staging as part of clinical history, the performance of detecting the historical status of TNM phrases was not evaluated in this paper and the rules were not applied.

S5. Evaluation of the CRC and TNM extraction algorithms

The CRC and TNM stage algorithms were evaluated at the report level: we judged whether a clinical report was correctly marked as describing current CRC, and whether the maximum TNM stage values were correctly extracted from the report (focus was on maximum values as some reports contained multiple staging scores).

Selection of clinical reports

To assess CRC detection, a hundred reports were randomly sampled from each of the four categories: imaging reports predicted to contain CRC according to the algorithm, imaging reports not predicted contain CRC, histopathology reports predicted to contain CRC, and histopathology reports not predicted to contain CRC. This was repeated separately for OUH reports used to develop the algorithm (selecting 400 reports), and on a newer sample of OUH reports unseen during development (selecting an additional 400 reports among these 'future reports'), yielding a total of 800 reports.

To evaluate TNM stage extraction, a hundred reports were similarly selected from each of the following categories: (1) imaging reports where a T, N or M value was detected; (2) imaging reports where no T/N/M values were detected; (3) histopathology reports where a T, N, or M value was detected; and (4) histopathology reports where no T/N/M values were detected. This process was again applied both to OUH reports used in algorithm development and to future OUH clinical reports, selecting a total of 400 + 400 = 800 reports.

The stratified random selection based on CRC and T/N/M detection helped ensure that enough positive samples were available to estimate the positive predictive value (PPV) and negative predictive value (NPV) of the algorithms, because many reports did not contain CRC or TNM staging. When sampling imaging reports, we only selected from reports that contained imaging types commonly used for investigating CRC and its metastases (for example, we included MRI scans of the pelvis but excluded x-rays of the foot) – a list of included imaging codes can be found at https://github.com/tammandres/crc-and-tnm .

The decision to select 100 reports from each category was based on feasibility (reviewing the reports is effortful and time of clinicians is limited), and on a simplified power analysis using Gaussian approximation to binomial distribution. According to the Gaussian approximation, when the true PPV of the algorithm is assumed to be 90%, selecting 100 reports ensures that there is only a 10% probability that the sample-based estimator differs from the true value by more than 5%.

We did not use clinical reports from the Royal Marsden (RMH) and Imperial College Healthcare (ICH) NHS Foundation Trusts to evaluate the algorithm at this stage. This is because it was not possible to obtain newer clinical reports from RMH as a future test set; and the ICH did not currently have enough resources to allow us evaluate the reports on their system. However, initial evaluation conducted on the RMH development set showed good performance, and examination of the extracted TNM phrases from ICH (without being able to view the left and right context) looked valid.

The OUH training data reports used in evaluation spanned December 2012 – December 2020, and the OUH test data reports spanned April 2022 – March 2023. The CRC and TNM extraction algorithms were previously applied to pathology and imaging reports of patients who had done the faecal immunochemical test (FIT) between 2017 and 2022 March. The algorithms' decision rules were most likely not updated then, but the date range of the future test data was nevertheless chosen to be from April 2022, so that it would be beyond the datacut of the FIT dataset, to ensure that the future test set would be independent from this previous run of the algorithms on FIT data.

Please note that one CRC report that had been sampled for evaluation was excluded from analysis, and hence the number of training data reports in Table 3 is 199 not 200. This is because the older report anonymisation pipeline that was used for that report had redacted a word, due to which a clinician could not determine whether the report discussed primary CRC or not. Report redaction did not interfere with establishing the ground truths for other reports.

Annotation of selected reports

The training data reports were initially reviewed by ND, and test data reports reviewed by AT and HJ (with cases that were not obvious assigned to HJ). After initial review, the annotations were further updated by AT and HJ to better account for cases where presence of colorectal cancer can be inferred from gene testing reports and where TNM stage can be inferred from report text but is not explicitly given. ND is a radiology registrar, HJ is a colorectal cancer surgeon, and AT is a data scientist with a molecular biology background. A specific annotation script was not used (other than requesting the clinicians to check that the presence of CRC and the TNM categories were correctly assigned by the algorithm). Due to restrictions on data access and limitations on clinician time, it was not practical for the same reports to be reviewed by multiple clinicians, and inter-annotator agreement was not computed. However, as the algorithms extract a limited number of categories with little subjective interpretation, we believe that re-annotation of the same dataset would have not provided substantial value given its time cost.

The algorithms were run on the selected reports and reviewed by displaying both the extracted values and reports in an R Shiny app (Figure S4). Ground truths were thus established by correcting extracted values. This was significantly more time effective than annotating reports from scratch, because 800 reports needed to be checked for 11 different TNM categories and another 800 for CRC status. Correcting extracted values risks inflating the algorithms' performance if incorrectly extracted or incorrectly missed values are accidentally overlooked, or if the annotator would be more likely to agree with the algorithms' prediction in borderline cases. The report viewer highlighted all patterns that resembled TNM stages (including false positives) and potential tumour keywords (including nonprimary tumours), making it less likely that values were missed. Error analysis showed that mismatches between algorithms' predictions and ground truth values were picked up. Reports where CRC status was not obvious were flagged for a review by a clinician and the clinician's notes demonstrated reasoning based on the report text rather than based on the algorithm's prediction (and it is unlikely that there is incentive to trust that algorithm above one's own reasoning, given that it is a regex-based tool and not a machine learning model). Furthermore, given that 11 TNM categories needed to be checked (each with multiple values), annotating 800 reports from scratch would risk unintentional data entry errors because an annotator would need to select values from a list many times and could plausibly click on the wrong value occasionally.

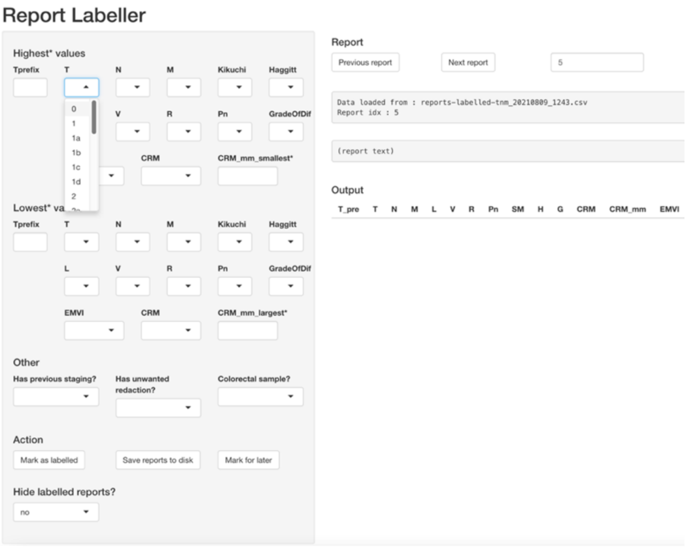

Figure S5. Shiny app for annotating imaging and pathology reports for TNM staging. The app displays reports and allows users to enter the required variables, or to correct information already extracted. It was originally developed for evaluating TNM stage extraction but can also be used for evaluating other types of extracted information.

Performance metrics
CRC and TNM stage extraction was evaluated with the metrics of PPV, NPV, sensitivity, and specificity, because these are familiar to clinical researchers. The outputs of the CRC detection algorithms were binary (a clinical report was predicted to discuss primary CRC or not), and hence the metrics could be computed in the usual way. However, as each TNM category can take multiple values, these metrics were computed relative to a 'null' value. For example, in the case of T category, PPV is the proportion of clinical reports where the maximum T value was correctly detected among all reports for which a T value was returned by the algorithm (i.e. where a non-null value was returned), NPV represents the proportion of reports that were correctly marked as not containing a T value among all reports where no T values were detected by the algorithm, sensitivity is the proportion of reports where the maximum T value was correctly detected among all reports that contained a T value, and specificity is the proportion of reports that were correctly marked as not containing a T value among all reports that did not contain a T value. PPV and sensitivity, when computed this way, are also known as micro-averages in the machine learning literature[5]. Macro-averages were not used because some TNM values (for example, “N2”) occurred much less frequently. For TNM staging, we additionally report the confusion matrix for all pairs of true and extracted values for a more detailed overview of performance.

The estimated sensitivity and specificity may not accurately represent the true sensitivity and specificity of the algorithm if it was run on all clinical reports available to us, because we used stratified random selection when sampling reports; however, they still indicate the proportion of positive and negative examples that were correctly detected in the random sample. The estimates of PPV and NPV, however, should reflect well the PPV and NPV of the algorithm on all clinical reports, given sufficient sample size.

S6. Errors of the CRC detection algorithm

Table S6. Errors of the CRC detection algorithm

Error category	Pathology reports	Imaging reports	Future pathology reports	Future imaging reports	Example
Reports describing gene testing not identified as CRC	23	-	33	-	“supplementary report … loss of MMR expression”
Tumour not seen	-	4	-	6	“tumour … hard to identify”
Treatment response	-	-	-	4	“no evidence of residual tumour”
Unclear tumour site	-	-	1	4	“changes in colon near the tumour”
General statement	-	-	-	3	“colorectal cancer MDT”
Falsely assigned non-CRC site	-	2	-	3	“chest/Pelvis imaging … tumour is identified in …”
Present marked as historic	-	2	-	3	“known tumour is … “
Historic marked as present	-	-	1	3	“patient had … tumour“
Possible marked as definite	-	3	2	1	“if the removed lesion was …”
Falsely assigned possible status	-	1	-	1	“possible to … <action> … tumour”
Benign mass	-	-	-	1	“no malignant features”
Describes metastasis not primary tumour	4	-	2	-	“liver … metastastic cancer … spread from colorectal”
CRC site missed	1	-	3	-	“transverse biopsy … cancer”
Summary does not state cancer	-	-	1	-	-
Small bowel cancer	-	1	-	-	-
No clear evidence of tumour	-	1	-	-	-
Recurrence not primary	-	1	-	-	-
Dysplasia not cancer	1	-	-	-	“colon tumour … dysplasia”
Falsely assigned CRC site	1	-	-	-	“anal margin … figo stage”
Number of falsely classified reports	*30*	*15*	*43*	*29*	
Total number of reports	*200*	*200*	*200*	*200*	

S7. Errors of the TNM stage extraction algorithm

Table S7. Errors for detecting T, N and M scores

TNM category	Error category	Count
**Pathology reports (training data)**		
T	implicit score X	39
T	implicit score 0	14
T	implicit score is	2
T	historic marked as present	2
N	implicit score X	1
N	historic marked as present	1
M	implicit score X	3
M	historic marked as present	1
**Imaging reports (training data)**		
T	implicit score X	14
T	implicit score 0	9
T	implicit score 4	5
T	historic marked as present	2
T	implicit score 4 and MRI sequence misclassified as T stage	1
T	undetected negation for single T value	1
N	implicit score X	3
N	implicit score 0	7
N	historic marked as present	2
M	implicit score 0	3
M	implicit score 1	5
M	implicit score X	10
M	M score missed due to regex pattern constraint	1
**Future pathology reports (test data)**		
T	implicit score X	15
T	implicit score 0	15
T	implicit score 1	3
T	historic marked as present	3
T	staging is not for colorectal cancer	3
**Future imaging reports (test data)**		
T	implicit score X	23
T	implicit score 0	13
T	implicit score 4	2
T	historic marked as present	4
T	MRI sequence misclassified as T stage	1
T	single value missed due to minimal context	1
T	subcategory missed due to spacing	1
N	implicit score X	2
N	implicit score 0	7
N	historic marked as present	1
M	implicit score 0	5
M	implicit score 1	4

Note. “implicit score” means that the staging could have been inferred from report text, but was not given in letters and numbers (e.g. inferring that there is no tumour ‘T0’ when the report says “colon – within normal limits”). “historic marked as present” means that historic staging was extracted from the report, and in these cases it was the only TNM staging available in the report.

S8. Simplified confusion matrix for the TNM staging algorithm

A simplified confusion matrix for the TNM staging algorithm is reported in Table S8. This matrix is 'simplified' because it ignores the subcategories of values (e.g. treats T1a, T1b, T1c, T1d as T1). This is to facilitate the examination of the algorithm (otherwise the table would be longer). Given that this matrix does not distinguish between TNM subcategories, the performance metrics computed based on this table do not exactly match the metrics reported in Table 4 of the main text. For example, if a T-stage was identified as T3a by the algorithm but T3b was correct, it was considered an error in Table 4, but not an error in the simplified confusion matrix because the main category (T3) was correctly detected.

Table S8. Simplified confusion matrix for detecting T, N and M scores

TNM category	True value	Predicted value	Count	Error
**Pathology reports (training data)**				
T	0	0	3	No
T	0	1	1	Yes
T	0	null	14	Yes
T	1	1	16	No
T	2	2	18	No
T	3	3	41	No
T	4	4	20	No
T	is	null	2	Yes
T	null	3	1	Yes
T	null	null	45	No
T	x	null	39	Yes
N	0	0	52	No
N	0	1	1	Yes
N	1	1	23	No
N	2	2	8	No
N	null	0	1	Yes
N	null	null	111	No
N	x	null	1	Yes
N	x	x	3	No
M	0	0	28	No
M	1	1	3	No
M	null	0	1	Yes
M	null	null	125	No
M	x	null	3	Yes
M	x	x	40	No
**Imaging reports (training data)**				
T	0	0	1	No
T	0	3	1	Yes
T	0	null	9	Yes
T	1	1	4	No
T	2	2	22	No
T	3	3	43	No
T	4	2	1	Yes
T	4	4	24	No
T	4	null	5	Yes
T	null	null	72	No
T	x	4	2	Yes
T	x	null	14	Yes
T	x	x	2	No
N	0	0	46	No
N	0	null	7	Yes
N	1	1	30	No
N	2	2	10	No
N	null	0	2	Yes
N	null	null	102	No
N	x	null	3	Yes
M	0	0	25	No
M	0	null	3	Yes
M	1	1	9	No
M	1	null	6	Yes
M	null	0	1	Yes
M	null	null	145	No
M	x	null	10	Yes

M	x	x	1	No
**Future pathology reports (test data)**				
T	0	0	2	No
T	0	1	3	Yes
T	0	null	15	Yes
T	1	1	18	No
T	1	null	3	Yes
T	2	2	14	No
T	3	3	41	No
T	4	4	18	No
T	is	is	1	No
T	null	1	2	Yes
T	null	2	1	Yes
T	null	null	67	No
T	x	null	15	Yes
N	0	0	45	No
N	1	1	23	No
N	2	2	9	No
N	null	null	121	No
N	x	x	2	No
M	1	1	3	No
M	null	null	192	No
M	x	x	5	No
**Future imaging reports (test data)**				
T	0	2	2	Yes
T	0	3	1	Yes
T	0	4	1	Yes
T	0	null	13	Yes
T	1	1	3	No
T	2	2	22	No
T	2	null	1	Yes
T	3	3	38	No
T	4	4	29	No
T	4	null	2	Yes
T	null	null	62	No
T	x	3	1	Yes
T	x	null	23	Yes
T	x	x	2	No
N	0	0	48	No
N	0	1	1	Yes
N	0	null	7	Yes
N	1	1	29	No
N	2	2	18	No
N	null	null	95	No
N	x	null	2	Yes
M	0	0	54	No
M	0	null	5	Yes
M	1	1	11	No
M	1	null	4	Yes
M	null	null	111	No
M	x	x	15	No

Note. The T, N and M scores can have subcategories, such as T2a and T2b. In this table, subcategories are ignored and grouped under the main category, for easier inspection of results (e.g. T1a and T1b are both treated as T1). Performance metrics computed in Table 6 of the main text however take into account the subcategories. Performance computed based on this confusion mi is highly similar to that shown in Table 6, but can be slightly higher as subcategory errors are not taken into account.

S9. Other TNM stage extraction algorithms

Several attempts have been made to extract explicitly given TNM staging values from free text (Supplementary Table S9). It is hard to evaluate how thorough and flexible these methods are as only two studies have publicly available source code [6,7] and one provides extraction rules in their publication [12].

Abedian et al [6,21] used a regex-based tool to extract TNM staging from pathology reports for four cancer subtypes. However, their regex requires the staging to be given after specific phrases, such as 'pathological staging', and allows for less variation in how the staging is written (does not allow gaps, mis-spelling of 0 as O, or repeated values). Odisho et al [7,22] also used regex, but required specific anchoring characters to precede the staging phrase ('pt', 'yp' and 'p') and seemed to require the staging to be given in a specific format 'pt<number>n<number>m<number>'. D'Avolio et al [12] allowed staging to be written more flexibly (gaps, 0 mis-spelt as O), but did not allow repeated values, text comments between TNM values, and TNM subcategories ('1a' instead of '1').

Ansoborlo et al [4] and Khor et al [8] note that they tried to account for false positive matches (such as 'T2' referring to a vertebra), and Khor et al [8] used exclusion keywords such as 'scan' to filter out these false positives, but it is not clear how flexible their extraction rules were otherwise. The other publications did not provide enough detail about the extraction rules [3,5,9–11].

Table S9. Studies where explicitly given TNM staging has been extracted from text

Author	Year	TNM categories	Source code	Cancer	Data	Train-test split	Region	Method	Flexibility
Ladas et al [3]	2023	preT, T, N, M, L, V, R, G, Pn	No	uknown	259 phrases from pathology reports	-	Germany	regex	unknown; evaluation of the TNM extraction was not the main focus of the paper
Ansoborlo et [4]	2023	T, N, M	No	tracheo bronchial	640 multidisciplinary team meeting reports	320 train, 320 test	France	regex, naive bayes classifier	unknown; accounts for false positive patterns (e.g. T2 vertebra)
Huang et al [5]	2023	T, N	No	urological	5404 pathology reports	4306 train, 1098 val, 268 test	Singapore	rule-based	unknown
Abedian et al [6]	2021	preT, T, N, M	Yes[13]	breast, colon, prostate, other	555,681 pathology reports	294 eval (unclear if any in train)	USA	regex applied to a window around anchoring term	limited: no gap, no misspelling, no repetition, strict prefix, requires anchoring phrase (such as "primary tumour")
Odisho et al [7]	2020	T, N, M, Pn	Yes[14]	prostate	3232 pathology reports	2066 train, 16% val, 10% test, 10% true test	USA		limited: requires anchoring characters ("pt", "yp", "p"), and format pt<number>n<number>m<number>
Khor et al [8]	2019	T, N, M	No	bladder, kidney, prostate, testes	1054 pathology reports, radiology reports and clinical notes	386 train, 668 test	Australia	regex	unknown; accounts for false positive patterns (e.g. T1 image)
AAlAbdulsalam et al [9]	2018	T, N, M	No	colon, lung, prostate	810 abstract records and pathology reports	405 train, 135 val, 270 test	USA	regex	unknown
Kim et al [10]	2014	T, N, M	No	prostate	pathology reports	100 eval (unclear if any in train)	USA	regex	unknown
Ashis et al [11]	2014	T, N, M	No	breast, lung, prostate	pathology reports	400 eval (unclear if any in train)	USA	regex	uknown
D'Avolio et al [12]	2008	T, N, M	Regex in paper[12]	prostate	pathology reports	60 train (for TNM), 676 eval	USA	regex	allows for gap, 0 mis-spelt as O (in N and M). Does not allow repeted

						(unclear if any in train)			values or comments between TNM values

S10. Potential machine learning approaches

The TNM scores could have been extracted using a generative large language model (LLM) or by training a machine learning (ML) model to classify text extracts. For the task of extracting TNM scores given in letters and numbers, it is unlikely that LLMs or classifiers would provide significant benefit over the regex-based algorithm here. This is because the TNM scores are reported in limited formats [1], which would not differ much across institutions and over time. These formats can be encoded in regular expressions, and false positives would usually be avoided as long as at least three TNM values occur in a sequence (which would be expected, given that T, N and M are the main categories of the staging system). It is unclear how well ML models would extract explicit TNM stages, although the models would likely require more computational resources, which may not be justified over a simpler regex strategy. An ML classifier could still be useful and more accurate for distinguishing very short TNM score sequences from false positives based on the surrounding context (e.g. "T1 NO" might not be a TNM stage if "NO" is the negation word in capital letters), and for distinguishing whether the TNM stage refers to a historic tumour or present tumour.

LLMs or text classifiers would likely be more valuable for inferring the TNM stage if it is not given in letters and numbers. With appropriate prompting, LLMs may be able to infer the staging without fine tuning while being robust to variations in language between hospitals, although their output may still need post-processing, and the user would not know how confident to be in the extracted data without an additional evaluation study. LLMs that are not open source may additionally require secure cloud environments to protect the private data in clinical reports, whereas open-source LLMs could be deployed locally with more control over the software given enough computational resources. However, it is possible that a smaller domain-specific bidirectional transformer-encoder model (BERT) [23], such as Bio+Clinical BERT [24], could be fine-tuned to perform the task sufficiently well with less compute. This could be achieved by fine-tuning the model to classify text extracts for each component of the TNM staging system (such as nodal status), and these components can be combined to infer the staging, as done in CanStaging+ [20]. If the classifier is calibrated, it would also provide meaningful confidence scores, allowing the less confident predictions to be reviewed.

Even though the CRC detection algorithm achieved high PPV (≥93.0), it could likely be improved by fine-tuning a BERT model to classify whether each of the short text extracts discussing CRC refers to current primary CRC. The text extracts themselves can be retrieved using the existing regex. A BERT type model can simultaneously consider both the left and right context of each phrase and potentially include more nuances than manually curated regex patterns can. The short phrases describing CRC would likely contain sufficient information for inferring whether CRC was primary, so generative LLMs that can reason over larger context windows may not be necessary.
